# Estrogen-Dependent Variation in the Contributions of TRPM4 and TRPM5 to Fat Taste

**DOI:** 10.3390/nu17243847

**Published:** 2025-12-10

**Authors:** Emeline Masterson, Naima S. Dahir, Ashley N. Calder, Yan Liu, Fangjun Lin, Timothy A. Gilbertson

**Affiliations:** 1 Burnett School of Biomedical Sciences, College of Medicine, University of Central Florida, Orlando, FL 32827, USA; emeline.masterson@ucf.edu (E.M.); ndahir@umich.edu (N.S.D.); ascalder@med.umich.edu (A.N.C.); fangjun.lin@swun.edu.cn (F.L.); 2 Department of Medicine, College of Medicine, University of Central Florida, Orlando, FL 32827, USA; liu.yan.7605@gmail.com

**Keywords:** taste, estrogen, TRPM4, TRPM5, fatty acid, sex differences, patch clamp, calcium imaging

## Abstract

Background: Sex differences in physiology have garnered significant interest of late; however, comparatively little is known about the effects of sex on the function of the peripheral taste system. Previously, we have shown that fat taste functions in a sexually dimorphic manner using molecular, cellular, and behavioral assays, and that a subtype of estrogen receptor (ER) proteins is highly expressed in Type II (receptor) cells. The underlying mechanisms of estrogen’s action, though, remain unknown. Objective: Here, we sought to better understand estrogen’s role in fat taste transduction at the molecular level by initially focusing on the transient receptor potential channel types M4 (*Trpm4*) and M5 (*Trpm5*), which we have shown to play roles in estrogen-sensitive fatty acid signaling in taste cells. Methods/Results: Using a multidisciplinary approach, using *Trpm5*-deficient mice, electrophysiological and calcium imaging assays revealed that there are significantly reduced FA responses in both males and females in the estrus phase, whereas females in the proestrus phase did not show this, suggesting that there may be E2-*dependent* TRPM5-*independent* FA signaling in Type II cells. During periods of high levels of circulating estrogen, there was no significant difference in cellular responses to fatty acid (FA) stimuli between *Trpm5*^−/−^ mice and their wild-type counterparts. Moreover, supplemental estradiol enhanced linoleic acid (LA)-induced TRPM5-mediated taste cell activation. Finally, while Type II cells depend on TRPM4 and TRPM5 for FA taste cell activation, proestrus (high-estrogen) females showed a greater dependence on a TRPM5-*independent* pathway for fatty acid responsiveness. Conclusions: Together, these results underscore the substantial regulatory role of estrogen in the taste system, particularly for fatty acid signaling. Given that the taste system guides food preferences and intake, these findings may have important implications for understanding sex-specific differences in diet and, ultimately, metabolic health.

## 1. Introduction

Males and females have long reported differences in ingestive behaviors and the development of metabolic disorders [1,2,3]. Women tend to have higher ratios of subcutaneous to visceral fat, as well as a higher prevalence of obesity [4,5,6]. Despite longstanding research highlighting these sex differences, the exploration of this phenomenon has been limited [3,7]. Investigations into sex-dependent variations in obesity and fat perception have uncovered distinct physiological responses. Specifically, Tanaka et al. (2022) observed a greater reduction in fat taste sensitivity in obese males relative to obese females [8]. Concurrently, Shi et al. (2022) demonstrated that, despite greater weight gain on a high-fat diet, females displayed a lower propensity for hepatic lipid accumulation than males, suggesting potential sex-specific differences in lipid metabolism and partitioning [2]. The consequences of obesity are also sex specific. Recent studies have shown that a high-fat diet exacerbated the cognitive decline in Alzheimer’s disease in females [9]. It is understandable, then, that the taste system, in its role of guiding food intake and dietary preferences, would also display sex differences. In fact, distinct differences in female taste palatability across numerous taste qualities have been demonstrated in both humans and murine models [10,11,12].

The incredible plasticity of the taste system in response to hormonal or dietary changes has been shown in recent studies. Highlighting the roles of hormones, specifically adiponectin and estradiol, in modulating taste receptor sensitivity [11,13]. Additionally, fat taste responsiveness is regulated in part by the sex hormone estradiol [14]. Demographic factors, such as age and sex, as well as physiological changes like obesity, also contribute to variations in taste perception [8,10,15]. This research underscored the plasticity of the taste system in response to sex hormones and highlights the need for further investigation into estrogen’s specific role in the taste system.

The taste system works as an essential bodily function, recognizing toxins and nutrients that guide our ingestive behaviors. This is accomplished through specialized taste cells that are embedded in our tongues, in taste buds that respond to tastants such as sweet, bitter, umami, salty, sour, and fat. Four distinct taste cell types are differentiated by their responsiveness to different taste stimuli: Type I (glial-like) cells, which may respond to amiloride-sensitive salty sensation, and Type II (taste receptor) cells that respond to sweet, bitter, umami, and fat stimuli. Type III (pre-synaptic) cells contain ion channels for sour taste and may have indirect responses to other tastants, and Type IV (basal/progenitor) cells [16]. The gustatory perception of fat is most commonly experienced through the consumption of dairy products and is exemplified by individual preferences for specific milk types (e.g., whole, 1–2% fat), indicating variations in particular sensitivity to fat stimuli [17]. In the early stages of fat taste, research highlighted the dependence on the palatability of food energy and lipid content [18]. Essential fatty acids (FAs) are both calorically dense and provide a high energy content during typical digestion compared to other proteins and carbohydrates [19]. Since these early studies, the recognition of fat as an essential taste, as the FAs stimulate taste cells, has gained momentum [20]. The rise in metabolic diseases has only added to the push for more research into the recognition, detection, transduction, and transmission of fat taste stimuli in taste cells [21]. Studies have indicated that fat taste stimuli are transduced via a G protein-coupled receptor-mediated pathway that activates second messenger cascades, much like other taste signaling cascades, such as sweet, bitter, and umami [16,22,23].

These investigations have identified several other important signaling molecules, including transient receptor potential melastatin (TRPM) channels, specifically TRPM4 and TRPM5, which are calcium-activated, monovalent cation channels [24]. TRPM5 has been well established as an integral part of the depolarization mechanism in taste cells in response to sweet, bitter, umami, and fat stimuli [25,26,27,28,29]. After its activation by binding Ca^2+^ at two binding sites, a conformational rearrangement opens the ion-conducting pore [30], inducing membrane depolarization and stimulating afferent nerve fibers in the taste system [25,26,27,28,29]. Previous taste research has shown that TRPM5 ablation results in a loss of preference for FAs [28,31], reduced sweet taste preference, and increased glucose tolerance [32,33]. The dynamic responses due to loss or damage of TRP channels have been a topic of exploration for some time.

These TRP channels are expressed in various tissues and have been linked to numerous metabolic diseases [34,35,36]. Previous studies have shown that other TRP channels exhibit sex differences in non-taste tissues; for example, TRPM2 provided neuroprotection against ischemia in males but not in females [37]. The upregulation of TRPV6 is mediated by estrogen [38]. Cholesterol, the precursor for steroid hormones, has been shown to directly regulate the function of TRPV4, TRPM3, and TRPM8 [38,39,40]. Female hormones, specifically, have also been shown to influence physiological functions via specific TRP channels; TRPV1 sensitization involves estradiol and estrous cycle-dependent nociception [41]. Progesterone P4 decreases thermosensitivity via TRPV1 [42]. E2 modulates thermoregulation via TRPM8 [43]. TRPM4 expression and function were tightly linked to the estrous cycle in the vomeronasal organ and regulated the relative strength of sensory signals in vomeronasal sensory neurons [44].

Previous studies identified the importance of TRPM5 in the FA-induced taste cell activation and downstream signaling in the Type II taste cells in males [45]. Briefly, long-chain unsaturated FAs interact with G-protein coupled receptor-120 (GPR120) that initiates a signaling cascade beginning with *α*-gustducin to trigger phospholipase C beta 2 (PLC*β*2) cleavage of phosphatidylinositol 4,5-bisphosphate (PIP_2_) to inositol 1,4,5-trisphosphate (IP_3_). IP_3_ is then free to stimulate Ca^2+^ release from the endoplasmic reticulum via the IP_3_R3 receptor. The rise in intracellular Ca^2+^ then activates TRPM5, inducing membrane depolarization and pannexin-1 ATP release to stimulate afferent nerve fibers [23,28]. Notably, the downstream fat taste signaling element, TRPM5, was shown to be sensitive to female sex hormones, as administration of E2 or P4 decreased TRPM5 mRNA expression [14]. Although we recently reported similar sex-dependent expression differences in TRPM4 in taste cells [14], the influence of other TRPM channels besides TRPM5 in the fat taste signaling pathway remains unanswered. Elucidating the precise mechanisms of these receptor-hormone interactions is essential for advancing research aimed at identifying effective interventions for the obesity pandemic [20]. Like many others, we recognize the importance of understanding these sexual disparities in taste and sought to better understand them at the molecular level in the present study.

Here, we highlight sex-specific reliance on the TRPM5-*dependent* FA signaling pathway and estrous cycle-dependent functional roles for TRPM4 and TRPM5. Using ex vivo whole-cell patch clamp recordings and functional calcium imaging in taste cells, we found that TRPM4 and TRPM5 are pivotal in both male and female fat taste signaling. However, their functional activity is synchronized to the female reproductive estrous cycle. Additionally, this cyclical regulation of fat taste via TRPM4 and TRPM5 is dependent upon the ovarian sex hormone, 17β-estradiol. Taken together, the regulation of TRPM4 and TRPM5 function by female hormones in taste cells reveals a previously unknown signaling pathway for studying sex differences in mammalian taste physiology.

## 2. Materials and Methods

### 2.1. Animals

All experiments involving animals were performed in accordance with the American Veterinary Medical Association guidelines and with approval from the Institutional Animal Care and Use Committee at the University of Central Florida. Experiments were performed on male and female estrus-cycled mice. All pups underwent genotyping via standard ear-punch procedures, followed by PCR and electrophoresis to confirm the desired transgenic strain before use. Phospholipase C*β*2, a marker for type II taste cells, and Green Fluorescent Protein (PLC*β*2-EGFP) transgenic mice were generously provided by Dr. Nirupa Chaudhari (University of Miami School of Medicine). Transient receptor potential melastatin 5 knockout mice (*Trpm5*^−/−^ stock #013068) [45] were purchased from the Jackson Laboratory and backcrossed with the C57BL/6J strain for at least five generations in our animal facility. Mice were maintained on a 12:12-h light-dark schedule and given *ad libitum* access to chow and water.

### 2.2. Assessment of Estrus Status

Standard estrus cycling was used to determine relative hormone levels based on cycle stage. To categorize females into high- and low-estrogen groups, those in the early phase of their cycle, during diestrus or proestrus, when estradiol levels are rising and have peaked, were designated the high-E2 group. Those in the later phases of their cycle, estrus or metestrus, when estradiol circulation plateaus, represented the low-E2 group. The standard vaginal lavage was utilized [14]. Briefly, cells from the vaginal canal were collected through plastic Pasteur pipettes prepped with a 0.9% NaCl solution, and the vaginal canal was flushed. The collected cells were used to create a wet smear for light microscopy to determine the cycle stage based on cell number and morphology. To best predict when each mouse would be in a particular phase, vaginal cytology was repeated daily at a consistent time point, and at least ten days before experimentation to ensure typical estrus cycling.

### 2.3. Solution and Reagents

Standard Tyrode’s solution contained (in mM) 140 NaCl, 5 KCl, 1 CaCl_2_, 1 MgCl_2_, 10 HEPES, 10 glucose, and 10 Na pyruvate; pH 7.40 adjusted with NaOH; 305–315 mOsm. The sodium-free Tyrode solution contained (in mM) 280 mannitol, 5 KCl, 1 CaCl_2_, 1 MgCl_2_, 10 HEPES, 10 glucose; pH 7.40 adjusted with TrisOH; 305–315 mOsm. Extracellular solutions in which the calcium concentration was changed (Ca^2+^-free or 0.25 mM CaCl_2_) (in mM) contained 140 NaCl, 5 KCl, 1.75–2 ethylene glycol-bis (β-aminoethyl)-N, N, N′, N′-tetraacetic acid (EGTA), 1 MgCl_2_, 10 HEPES, 10 glucose, and 10 Na pyruvate; pH 7.40 adjusted with NaOH; 305–315 mosM. A potassium-based intracellular solution was used for the measurement of membrane potential and contained (in mM) 140 K gluconate, 1 CaCl_2_, 2 MgCl_2_, 10 HEPES, 11 EGTA, 1.5 ATP, and 0.5 GTP; pH 7.2 adjusted with KOH; 290–300 mOsm. Unless otherwise noted, all chemicals were purchased from Sigma Chemical Co. (St. Louis, MO, USA). 17β-estradiol (10 nM E2) was prepared in 100% ethanol; working concentrations were made from stock on the day of the experiment and dissolved in standard Tyrode’s, as previously shown to be effective [14]. Linoleic acid (LA) is a polyunsaturated fatty acid (PUFA) that has been considered the prototypical stimulus for FA taste and representative of other PUFAs [46]. LA was prepared as described previously [47]. Briefly, LA was prepared as a stock solution (25 mg/mL) in 100% ethanol, stored under nitrogen at −20 °C, and a working concentration (30 µM) was made immediately before the experiment. LA was dissolved in standard Tyrode’s in all experiments. An antagonist of TRPM5, triphenylphosphine oxide (TPPO; 100 µM), and an antagonist of TRPM4, 9-phenanthrol (9-PHE; 100 μM), were made immediately before the experiment. TPPO and 9-PHE were dissolved in dimethyl sulfoxide (DMSO), and the final concentration of DMSO was less than 0.1%. DMSO at the concentrations used here did not affect cell health or cellular responses, but was included in the LA-alone solutions.

### 2.4. Taste Cell Isolation

Individual taste cells were isolated from the tongues of 5 to 12-month-old mice as previously described [13,14,22,28,48]. Briefly, mice were euthanized with CO_2_ exposure and subsequent cervical dislocation, after which their tongues were promptly resected. The tongues were then injected between the muscle and lingual epithelial layers with an enzyme cocktail containing ~0.2 mL of Tyrode’s solution, collagenase A (0.5 mg/mL; Sigma, St. Louis, MO, USA), dispase II (2.0 mg/mL; Sigma, St. Louis, MO, USA) and trypsin inhibitor (1 mg/mL; Sigma, St. Louis, MO, USA). The latter was included to prevent undesirable proteolysis of membrane-bound proteins. The injected tongues were then incubated in a Tyrode’s bath with bubbled O_2_ for 30 min, to allow the enzyme cocktail to separate the muscle from the lingual epithelial layer, after which the lingual epithelium can be manually removed from the muscle layer and pinned out with the mucosal surface down on a Sylgard™-lined Petri dish. This epithelium was then incubated in divalent cation-free (Ca^2+^- and Mg^2+^-free) Tyrode’s solution containing 2 mM 1,2-bis(o-aminophenoxy)ethane-N, N, N′, N′-tetraacetic acid (BAPTA), followed by a second incubation of the enzyme cocktail at room temperature. For calcium imaging experiments in which individual taste cells are preferred, the epithelium was incubated in Ca^2+^- and Mg^2+^-free Tyrode’s for 4 min, followed by 2 min in the enzyme cocktail. For patch clamp experiments in which intact taste buds are preferred, the epithelium was incubated in Ca^2+^ and Mg^2+^-free Tyrode’s for 2 min, followed by 1 min of incubation with the enzyme cocktail. After incubation, taste buds or cells were removed by gentle suction using a ~100–150 µm fire-polished pipette under a low-magnification dissecting microscope. Isolated cells were plated onto 15 mm glass coverslips coated with Corning^®^ Cell-Tak™ Cell and Tissue Adhesive (Corning, NY, USA: PN 354240) for calcium imaging. Isolated buds were plated onto a Cell-Tak™ coated Superfrost microscope slide fitted with an O-ring fabricated from Sylgard™ 184 elastomer (Dow Chemical, Midland, MI, USA).

### 2.5. Patch Clamp Recording

The prepared slides containing taste cells were continuously perfused with a standard Tyrode solution at room temperature (RT) at a flow rate of 3.5 mL/min, as standardized in previous experiments [13,14,22,28,48]. Whole-cell recordings were obtained from taste cells maintained in taste buds at RT. Patch pipettes were pulled on a P-1000 Flaming/Brown micropipette puller (Sutter Instrument, Novato, CA, USA) from 1.5-mm outer-diameter filamented borosilicate glass tubing, which were then fire-polished to create resistances ranging from 6 to 10 MΩ. Membrane voltage and current signals were generated using an Axopatch 200 B and digitized at 10 kHz using a Digidata 1550 B and Clampex software 11.1 (Molecular Devices, San Jose, CA, USA). For membrane potential measurements from taste cells, the amplifier was operated in current-clamp mode while holding the cell at its zero-current level (i.e., at rest). This mode allows for recording depolarizations in the membrane potential, eliciting downstream nerve responses. In contrast, the voltage-clamp mode, which was used to measure taste cell currents in response to stimuli, allows recording of ion flux into the cell. Inward currents were recorded at a holding potential of −100 mV to prevent voltage-gated conductances in taste cells. By using both modes, we were able to clearly observe ion channel activity in these taste cells in response to fat stimuli. A 5-s stimulus (5–10 psi) was applied focally to the entire taste cell from a double-barreled theta-glass pipette (Warner Instruments, Holliston, MA, USA) positioned near the cell and delivered by a PicoSpritzer III (Parker Hannifin Corp., Cleveland, OH, USA) to obtain responses from the same cell. For single-stimulus applications, a micropipette filled with the appropriate stimulus was connected to the PicoSpritzer III and used to deliver a 5-s stimulus. Duration of stimulus application was standardized from prior experimentation [13,14,22,28,48]. Responses of taste cells were recorded continuously before, during, and after LA application, controlled by the data acquisition software. Response differences to FAs and other stimuli between single taste cells and taste cells contained in a taste bud showed no significant differences, and data were pooled accordingly.

### 2.6. Calcium Imaging

Ratiometric calcium imaging experiments utilizing Fura2-AM were conducted as previously described [13,14,22]. Briefly, prepared coverslips with isolated cells were loaded with intracellular calcium indicator Fura2-AM (Invitrogen, Waltham, MA, USA) for an hour of incubation before experimentation. To record changes in [Ca^2+^]_i_ cells were excited at 340 and 380 nm, and emission was recorded at 510 nm using the InCyt Im2 software (Intracellular Imaging Inc., Cincinnati, OH, USA). To identify the subset of Type II taste cells, the GFP-PLC*β*2 transgenic lines were utilized, then cells were excited at 490 nm to excite the EGFP-expressing cells, and emission was recorded at 510 nm. The GFP-expressing cells were marked within the software prior to recording the Fura-2 signal. The F340/F380 ratio of each cell was converted to [Ca^2+^]_i_ based on the calcium calibration kit (Invitrogen, Waltham, MA, USA). Prior to experimentation for this study, robust trials were performed to determine the most consistent responsive stimulus for isolated TBCs were performed and it was determined that linoleic acid (LA; 30 µM) would act as our standard fat stimulus. For these experiments, LA alone and a mixture of LA with TRPM4 and TRPM5 blockers, 9-PHE, and TPPO, respectively, in random order, were perfused over taste cells at a flow rate of 4 mL/min, followed by 1 min of 0.1% fatty acid-free BSA solution as previously described [13,14,22,28,48]. After each application, Tyrode’s solution was applied until the calcium signal returned to baseline before the next solution was applied.

### 2.7. Immunofluorescence Staining

Male and female GFP-PLC*β*2 transgenic mice were deeply anesthetized with isoflurane before transcardiac perfusion, first with saline, followed by a 4% paraformaldehyde (PFA) solution in 0.1 M phosphate buffer (pH 7.4). After this, the tongues were removed and postfixed in PFA for 2 h, followed by a 30% sucrose solution in 0.1 M PBS (pH 7.4) overnight at 4 °C. Tongues were then separated into anterior sections containing the fungiform papillae and posterior sections containing the circumvallate papillae, which were embedded in the OCT compound (Tissue-Tek^®^). Frozen coronal sections were cut at 20 μm using a cryostat and mounted onto Superfrost glass slides. Sections were washed with 0.1 M PBS, and nonspecific binding was blocked for 1 h in a blocking solution (10% serum, 0.3% Triton X-100 in PBS). Primary antibodies, KO validated by the vendor [1:100 anti-TRPM4, Alomone Labs, Jerusalem, Israel (ACC-044)], were diluted in blocking solution and applied to sections for incubation overnight at room temperature. After rinsing in PBS, the sections were incubated with 1:250-diluted goat anti-rabbit Alexa Fluor 594 (Invitrogen, Waltham, MA, USA) for 2 h. After washing with PBS, nuclei were stained with 0.5 mg/mL Hoechst 33,258 in 0.1% PBS-Tween^®^ 20 (PBST) for 10 min at room temperature. The sections were rinsed 3 times in PBS, and coverslips were mounted with Fluoromount G (Southern Biotech, Birmingham, AL, USA). Negative controls were obtained by omitting the primary antibodies only to assess any background signal. Sections were imaged with a laser scanning confocal microscope (Zeiss, LSM710, Oberkochen, Germany) equipped with 405, 488, 561, and 633 laser lines. Images were processed and analyzed with ImageJ (ImageJ 1.50, National Institutes of Health, Bethesda, MD, USA).

### 2.8. Statistical Analysis

Though linoleic acid was used as the prototypical and representative PUFA, to further characterize this fatty acid prior to experimentation, dose–response relationships in taste receptor cells (TRCs), half-maximal effective concentrations (EC_50_) were determined by fitting four-parameter logistic dose–response functions to model-derived response estimates [46]. Group differences were further examined using two-way analysis of variance (ANOVA) followed by Bonferroni’s post hoc tests for multiple comparisons. Similarly, to evaluate estradiol (E2) sensitivity in TRCs prior to experimentation, calcium responses were quantified as area under the curve (AUC) and compared across experimental groups using a two-way ANOVA with Bonferroni’s post hoc multiple-comparison procedures.

For all experiments, *n* = 1 represents a singular taste cell; singular cells were collected from multiple animals from a minimum of three independent experiments conducted on different days. Data were gathered from experimental software, InCyt Im2 software (Intracellular Imaging Inc., Cincinnati, OH, USA) and Clampex software (Molecular Devices, San Jose, CA, USA) for calcium imaging and patching, respectively. These traces were then baseline-corrected in Origin 8–9.6 (OriginLab, Northampton, MA, USA) and transferred for statistical analysis in GraphPad Prism version 8.0 (GraphPad Software Inc., San Diego, CA, USA), as described in the experiment below.

## 3. Results

### 3.1. TRPM5 Is Nonessential for LA-Induced Type II Taste Cell Activation in Early-Phase Estrus Cycle Females

**Figure 1:**
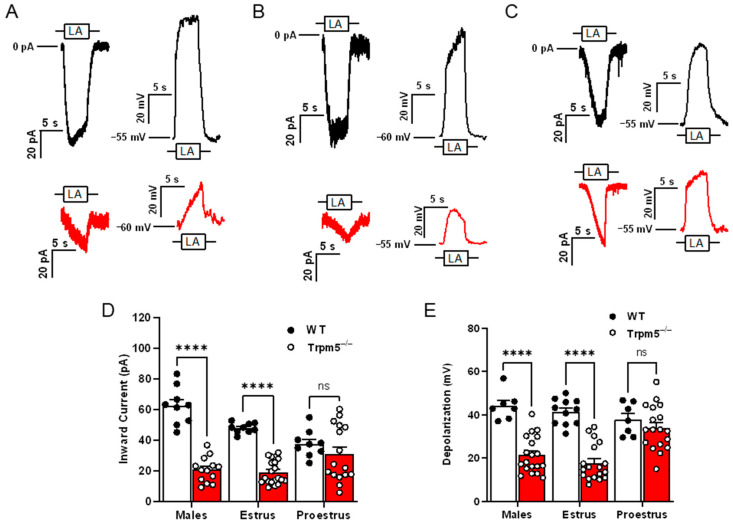
TRPM5 is nonessential for LA-induced Type II taste cell activation in early-phase estrus cycle females. Representative 30 µM linoleic acid (LA)-induced currents and membrane depolarization in wild type (black traces) and *Trpm5*^−/−^ mice (red traces) in males (**A**), females in estrus (**B**), and females in proestrus (**C**). Shown are traces of whole-cell *Trpm5*^−/−^ currents generated by focal application of LA (30 µM) through a 5-s application via a stimulus pipette. Inward transient currents (at V_h_ = −100 mV) and changes in V_MEM_ (at zero current potential) were recorded from wild-type (PLC*β*2-GFP) mice and *Trpm5*^−/−^ mice. (**D**) Cumulative graphs of inward current transients in response to LA in male, female mice in estrus and female mice in proestrus phases of the estrous cycle. Error bars indicate SEM across cells; *n* = 9 cells for wildtype and *n* = 13–20 cells from *Trpm5*^−/−^ mice. (**E**) Cumulative graphs of traces depicting total cellular depolarization in response to rapid application of LA (30 µM). Cellular depolarization was recorded from wild-type (PLC*β*2-GFP) and *Trpm5*^−/−^ mice at resting membrane potential (V_MEM_ = −55 to −60 mV). Error bars indicate SEM across cells; *n* = 7–11 cells from wild type and *n* = 18–19 cells from *Trpm5*^−/−^ mice. **** *p* < 0.0001; ns, not significant.

Two-way ANOVA with Bonferroni’s method for post hoc multiple comparisons was performed to assess the sex differences as well as the effects of the estrus cycle on TRPM5-mediated FA signaling (inward current interaction, F(2,70) = 14.2, *p* < 0.0001; cellular depolarization interaction, F(2,74) = 9.507, *p* < 0.001). It had been anticipated that in cells isolated from male *Trpm5*^−/−^ mice, a significant reduction in response to LA-induced inward current and cellular depolarization would emerge when compared to male wild-type mice (Figure 1A,D,E; *p* < 0.001). Similarly, the late-phase females with low levels of circulating estrogen also experienced a reduction in response in the *Trpm*5^−/−^ mice compared to wildtype (Figure 1B,D,E; *p* < 0.001). Unexpectedly, we found that in early-phase females, which are exposed to high levels of circulating endogenous estrogen, FA responses were not significantly reduced in the *Trpm5*^−/−^ mice compared to their wild-type counterparts (*p* = 0.4812).

### 3.2. In Type II Cells, Estradiol Increases LA-Induced Taste Cell Activation of the TRPM5-Mediated Pathway

**Figure 2:**
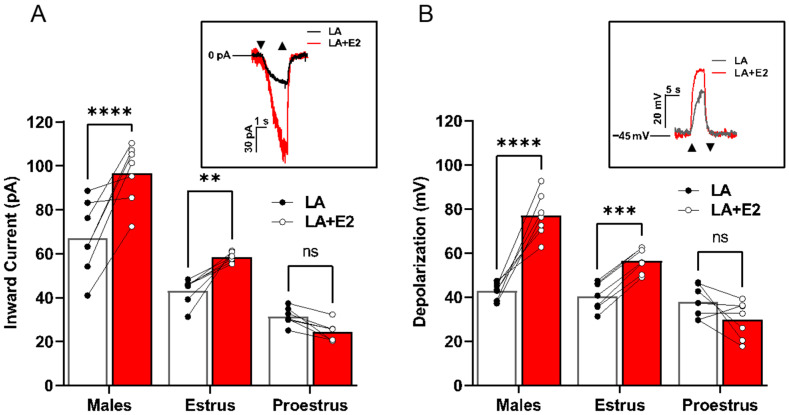
Estradiol increases Type II cell activation in a sex-dependent manner. (**A**) Males and females during the estrus phase of the estrous cycle display an increase in Type II taste cell inward current when FAs were combined with E2 compared to FAs alone. The same cells were used for both stimuli to examine the E2 effect (*n* = 7 cells for each group). Inset, example inward current trace from a male Type II taste cell that is activated by estradiol with LA + E2 or LA alone. (**B**) Total cellular depolarization is increased in males and females during the estrus phase of the estrous cycle when Type II taste cells are activated by LA with E2 compared to LA alone (holding potential (V_h_) = −100 mV). The same cells were used for both stimuli to examine the E2 effect (*n* = 7 cells for each group). Inset, example traces of the total cellular depolarization from a male Type II taste cell that is activated with LA with E2 or LA alone (resting membrane potential (V_MEM_) = −45 mV. Cells from females in proestrus showed no enhancement of LA-induced currents or V_MEM_ changes by E2 treatment (*n* = 6 cells per group). Triangles show regions of stimulus onset and termination. **** *p* < 0.0001; *** *p* < 0.001; **, *p* < 0.01; ns, not significant.

LA-induced inward currents (Figure 2A) and total cellular depolarization (Figure 2B) were recorded from Type II taste cells in the presence or absence of exogenous E2. A two-way ANOVA with the post hoc Bonferroni’s method revealed significant sex- and estrus cycle-dependent differences in taste cell responses to LA (inward current interaction, F(2,18) = 18.05, *p* < 0.001; cellular depolarization interaction, F(2,18) = 34.59, *p* < 0.001). Cells from early-phase females showed no significant difference in LA-induced inward currents (*p* = 0.3677). In contrast, Type II cells from both males and late-phase females had significantly increased inward currents (males *p* < 0.001, late-phase females *p* = 0.0074) and total cellular depolarization (males *p* < 0.001, late-phase females *p* = 0.0009) in response to LA with added E2 (Figure 2B; V_mem_ 49.57 ± 3.81). In a manner analogous to the loss of TRPM5, early-phase female responses to LA were insensitive to E2-induced increases in taste cell activation, as seen in males and late-phase females (*p* = 0.1097).

### 3.3. TRPM4 Is an Essential Part of LA-Induced Fatty Acid Signaling in Type II Cells

**Figure 3:**
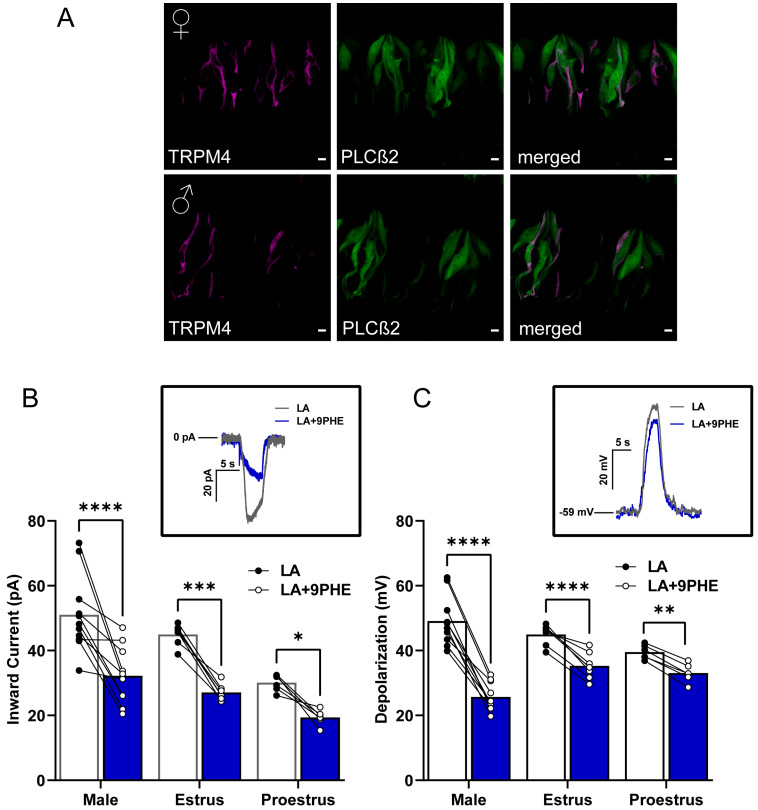
TRPM4 is a key component of fatty acid signaling in Type II cells from male and female mice. (**A**) Immunofluorescence staining from left to right panel showing TRPM4 (magenta), PLC*β*2-GFP (green), and merged image. Scale bars represent 5 μm. (**B**) LA-induced inward current in Type II cells is significantly reduced by the TRPM4 channel antagonist 9-phenanthrol (9 PHE) in males and females across the estrous cycle. The same cells were rapidly exposed to LA and LA with TRPM4 channel antagonist, 9-phenanthrol (*n* = 11 cells for males and *n* = 6–7 cells for females). Inset, inward current trace from male Type II taste cell that is activated by LA or LA with TRPM4 channel inhibitor 100 µM 9-phenanthrol: holding potential (V_h_ = −100 mV). (**C**) Total cellular depolarization induced by LA in Type II taste cells is significantly reduced by the TRPM4 channel inhibitor, 9-phenanthrol, in males and females during the estrus phase but not in females during proestrus of the estrous cycle (*n* = 10 cells for males and *n* = 6–7 cells for females). Inset, Cellular depolarization of Type II cell rapidly stimulated with LA and LA with the TRPM4 channel inhibitor (100 µM 9-PHE); resting membrane potential. (V_MEM_) = −59 mV). **** *p* < 0.0001; *** *p* < 0.001; ** *p* < 0.01; * *p* < 0.05.

To begin, we needed to confirm the colocalization of our PLC*β*2-GFP Type II cell marker with TRPM4 proteins via immunofluorescence. We confirmed the presence of TRPM4 protein in the Type II taste cells of male and female mice (Figure 3A). After confirming the presence of TRPM4 in the Type II cells, we sought to confirm its functionality in these LA-mediated pathways in taste cells. To do so, we utilized 9-phenanthrol (9-PHE), a selective TRPM4 antagonist, in males and females and compared the LA-induced Type II taste cell activation. Two-way ANOVA with Bonferroni’s method for post hoc multiple comparisons was performed. 100 μM of 9-PHE significantly reduced the LA-induced inward currents in Type II cells across all groups (inward current drug interaction, F(1,21) = 60.95, *p* < 0.0001; depolarization drug interaction, F(1,20) = 176.9, *p* < 0.0001). Similarly, the total cellular depolarizations from males and late-phase females showed significantly decreased responses to LA, with inhibition of TRPM4 (males *p* < 0.001, late-phase females *p* < 0.001). Consistent with our previous observations, the early-phase females exhibited a smaller significant reduction in total cellular depolarization response following the loss of TRPM4 as compared to their counterparts (*p* = 0.0082).

### 3.4. Type II Taste Cells Utilize Both TRPM4 and TRPM5 for LA-Induced Taste Cell Activation

**Figure 4:**
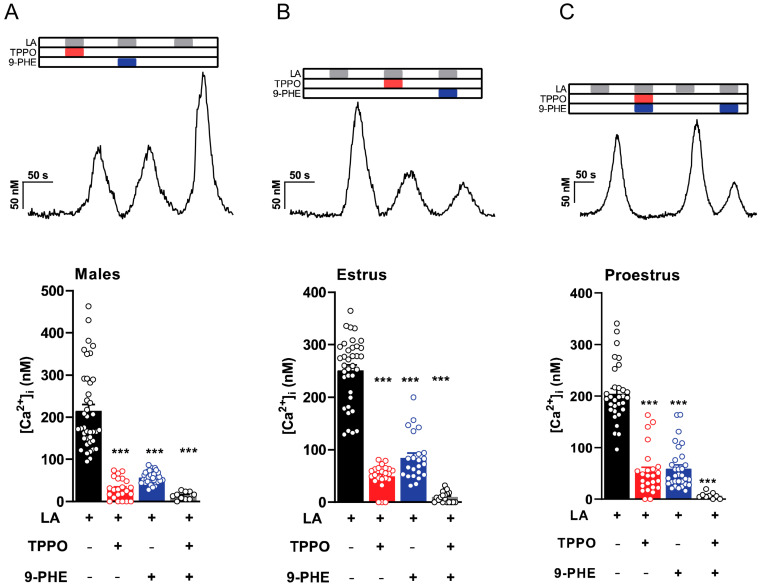
Both TRPM4 and TRPM5 antagonists inhibit intracellular calcium rise in Type II taste cells. Representative calcium traces depicting effects of TRPM4 and TRPM5 blockers on LA-induced taste cell responses from Type II taste cells in response to LA, LA with TRPM5 channel inhibitor (100 µM triphenylphosphine oxide [TPPO]), LA with TRPM4 channel inhibitor 100 µM 9-phenanthrol (9 PHE), or LA with both TRPM4 and TRPM5 channel inhibitors in males (**A**), females in estrus (**B**), and females in proestrus (**C**). Summary data of intracellular calcium responses induced by LA in the presence or absence of TRPM4 and TRPM5 channel inhibitors are shown from the same group below. TRPM4 and TRPM5 channel inhibitors decreased LA-induced intracellular calcium concentration in Type II taste cells (error bars indicate SEM across cells; *n* = 23–34 cells from males, *n* = 23 cells from females on estrus, and *n* = 25–31 cells from females on proestrus). *** *p* < 0.001.

To further evaluate the roles of TRPM4 and TRPM5 in the Type II cell FA signaling pathway, we used selective antagonists, TPPO for TRPM5 and 9-PHE for TRPM4, to inhibit them and assess responses. We performed calcium imaging on single-taste cells isolated from PLC*β*2-GFP mice loaded with FURA-2 AM, a radiometric fluorescent dye, and measured the LA-induced intracellular calcium responses. A two-way ANOVA with Bonferroni’s method for post hoc multiple comparisons was performed to compare responses with and without the inhibitors (males: F(3,64) = 67.96, *p* < 0.0001; estrus: F(3,63) = 201.6, *p* < 0.0001; proestrus: F(3,62) = 132.6, *p* < 0.0001). These results indicated that TRPM4 (9-PHE) and TRPM5 (TPPO) antagonists inhibited LA-induced calcium responses, and the combination of both channel antagonists markedly abolished these responses in both male and female mice (Figure 4A–C). These results indicated that LA-induced calcium activation requires TRPM4 and TRPM5, suggesting an essential role in the inward current response of the taste cells irrespective of sex. As expected, the inhibition of TRPM4 and TRPM5 caused a complete abrogation of calcium responses in the Type II cells. Interestingly, however, the relative effects of the loss of either TRPM4 or TRPM5 varied across the estrus cycle.

### 3.5. Early-Phase Females Demonstrate Enhanced Utilization of the TRPM5 Independent Pathway

**Figure 5:**
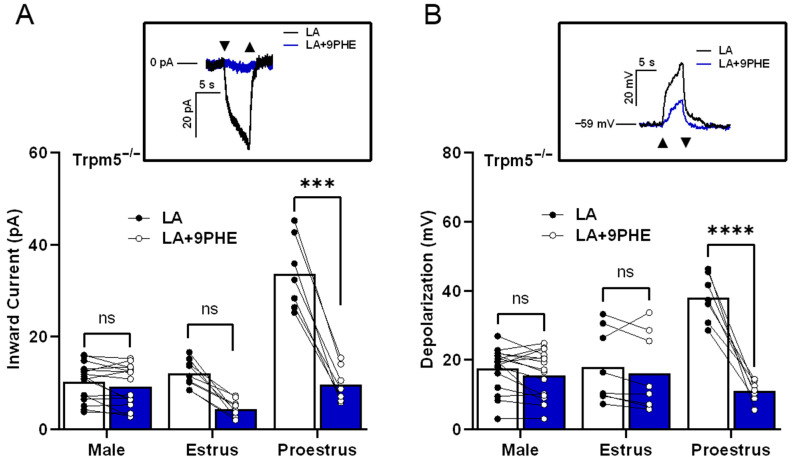
In the absence of normal functioning TRPM5, females in proestrus utilize TRPM4 for LA-induced taste cell activation. LA-induced inward current in female *Trpm5*^−/−^ mice during the proestrus phase requires TRPM4. (**A**) Inward current from *TRPM5*^−/−^ mice stimulated with LA in the presence or absence of TRPM4 channel inhibitor, 100 µM 9-phenanthrol (9 PHE; *n* = 16 cells in males, *n* = 7–9 cells in female mice). Inset, an example of inward current traces in a *Trpm5*^−/−^ female taste cell during the proestrus phase that is stimulated with LA and LA with 9-phenanthrol, holding potential (V_h_ = −100 mV). (**B**) Total cell depolarization from *Trpm5*^−/−^ mice exposed to LA and LA with 9-phenanthrol. In the absence of TRPM5, TRPM4-mediated cellular depolarization is essential in females during proestrus phases (*n* = 16 cells in males, *n* = 7–9 cells in female mice). Inset, an example of cell depolarization traces in a *Trpm5*^−/−^ female taste cell during the proestrus phase that is stimulated with LA and LA with 9-phenanthrol, resting membrane potential (V_MEM_) = −59 mV. Triangles show regions of stimulus onset and termination. **** *p* < 0.0001; *** *p* < 0.001; ns, not significant.

To test the ability of early-phase females to utilize a TRPM5-*independent* pathway, we used *Trpm5* knockout mice and the TRPM4 antagonist 9-PHE. Two-way ANOVA with Bonferroni’s method for post hoc multiple comparisons was performed. We found that the remaining inward current in the *Trpm5*^−/−^ males and females in the estrus phase showed no significant difference from the loss of *Trpm5* in the presence or absence of TRPM4 inhibitors (*p* > 0.999, estrus: *p* = 0.133). However, the LA-induced inward current from females in the proestrus phase significantly decreased when TRPM4 function was inhibited in addition to the loss of *Trpm5*, as shown in Figure 5A (proestrus: *p* < 0.0001). Similar to the effects in LA-induced inward currents, the LA-induced total cellular depolarization was only affected by TRPM4 inhibition in the females during the proestrus phase, as shown in Figure 5B (males: *p* > 0.999, estrus: *p* > 0.999, proestrus: *p* < 0.0001). These results indicate that LA activation in taste cells shows sex- and estrus-cycle-dependent differences in TRPM4′s role, and support a functional role for TRPM4 in *Trpm5*^−/−^ mice.

## 4. Discussion

### 4.1. TRPM5 Is Nonessential for LA-Induced Type II Taste Cell Activation in Early-Phase Estrus Cycle Females

There have been few, if any, studies that have characterized sex differences in distinct cell types within the peripheral taste system. We provide the initial electrophysiological evidence of sex differences in response to fatty acids. Patch-clamp recordings were made from Type II taste cells, which were unambiguously identified by their intrinsic fluorescence in mice expressing GFP under the control of PLCβ2 [49].

Previous work also demonstrated the significant role that TRPM5 played in the Type II taste cell response to linoleic acid taste perception in male mice [25,28]. Our previous studies examining the effects of sex on the plasticity of fat-taste signaling pathways revealed sex-dependent *Trpm5* expression in taste cells. These differences persisted in females throughout the estrus cycle, with significant fluctuations between early and late estrus phases [14]. In combination, these studies revealed a gap prompting our investigation into the functional role of TRPM5 in mediating sex differences in fat taste. To assess the role of TRPM5, we utilized a knockout model lacking systemic *Trpm5*. In contrast to an earlier study [29], we demonstrated the presence of TRPM4 and IP_3_R3 using PCR, immunohistochemistry, and functional antagonism with a specific pharmacological agent (see Supplementary Figure S1). By combining differential contrast imaging with immunohistochemical staining for TRPM4 and IP_3_R3, we validated their presence in the *Trpm5* knockout mice (Supplementary Figure S1A,D). Along with *Trpm5*^−/−^ male and normal cycling female *Trpm5*^−/−^ mice, we also utilized taste cells from our PLC*β*2-GFP transgenic mice, which specifically highlight Type II cells.

### 4.2. Relative Contributions of TRPM4 and TRPM5 Vary Across the Estrous Cycle

Similar to previous experiments, we found that Type II cells from male mice required TRPM5 for FA activation (see Figure 1). In electrophysiological experiments, both the TRPM5-mediated inward currents and cellular depolarizations were significantly reduced in male *Trpm5*^−/−^ mice. The minor discrepancies between the inward current and depolarization measurements demonstrate the complementary nature of these two techniques. While the inward current measurement quantifies the flow of positively charged ions (such as sodium) into a cell, an inward current initiates cellular depolarization only after a sufficient influx of ions significantly alters the membrane potential. By concurrently measuring cellular depolarization, reflected by a shift in membrane potential towards a more positive value, we can directly observe the relationship between ion flux and cellular activation.

In contrast to males and the majority of earlier experiments, we found that TRPM5 was not solely responsible for taste cell activation in female mice and that TRPM5′s contribution to FA-induced taste cell activation was highly dependent on the estrous cycle [23,25,26,27,28,29]. To investigate the role of female cyclic hormones in this TRPM5 FA signaling pathway, these experiments utilized the estrous cycle as a proxy for hormone levels, specifically estradiol (E2). E2 levels exhibit a cyclical pattern, declining during the late estrus phase and rising during the early proestrus phase [50,51]. For example, females in the later estrus phase of their cycle, during which their circulating E2 levels are lower and more similar in magnitude to those of males, showed a significant decrease in both TRPM5-mediated inward currents and cellular depolarizations. However, females in the early proestrus phase showed no significant electrophysiological difference in the FA-induced response between wild-type and *Trpm5* knockout mice. This continued response in the absence of TRPM5 in early-phase females indicates a role for cyclic ovarian hormones in fat taste signaling in female mice. The level of endogenous E2 may influence the use of the TRPM5-mediated fat-signaling pathways. Together, these results support the idea of a TRPM5-*dependent* pathway for fat taste and that this pathway is both sex and estrus cycle-dependent, as females during high endogenous E2 do not require TRPM5 for LA-induced taste cell activation.

### 4.3. In Type II Cells, Estradiol Increases LA-Induced Taste Cell Activation of the TRPM5-Mediated Pathway

Our data suggested a significant role for circulating ovarian hormones in the sex differences seen in taste cell activation. Previous work supported this notion as estradiol increased LA-induced intracellular calcium responses in Type II taste cells [14]. To further explore the role of E2 in the TRPM5-mediated fat-signaling pathway, we assessed the acute effects of E2 in TRPM5-mediated FA taste activation. Previous research had shown that the taste cells did not respond to E2 stimulation alone. However, when applied in combination with FA stimuli (LA + E2), it produced a significant increase in calcium signaling compared to LA alone in taste cells from males and females with low endogenous estrogen [14]. We further examined the interaction between TRPM5-mediated taste cell activation and endogenous E2 levels in females. The experiments showed that the focal application of LA combined with E2 significantly increased both the TRPM5-mediated inward currents and depolarization in wild-type and knockout males, as well as in wild-type and knockout estrus phase females, when compared to LA alone (see Figure 2). Interestingly, there were no significant E2-induced alterations between the wild-type and knockout inward currents and cellular depolarizations observed in females in the proestrus (high E2) phase. Although not significant, the currents in wild-type females during the proestrus phase had a slightly smaller observable magnitude than either the wild-type males or females in the estrus phase. These data indicate that the influence of estradiol on the TRPM5-mediated FA signaling pathway is not only sex-dependent but also estrous-cycle-dependent as well. These findings, highlighting a role for cyclic ovarian hormones in TRPM5 signaling, should be explored in other systems, for similar responses could provide novel mechanisms for numerous therapies [31,32,33,34]. However, the absence of significant differences in either wild-type or knock-out proestrus females suggests that another mechanism carries the LA-evoked inward movement of Na^+^ ions that promotes taste cell activation.

### 4.4. TRPM4 Is an Essential Part of LA-Induced Type II Cell Fatty Acid Signaling

TRPM4 has frequently been proposed to act in concert with TRPM5 to depolarize Type II taste cells in response to sweet, bitter, and umami tastants [16,23,29]. Additionally, our previous work on the *Trpm5* gene expression showed that *Trpm4* expression varied across sexes and the estrus cycle [14]. Given that females during the early proestrus phase of the estrous cycle were able to utilize a TRPM5*-independent* pathway more than their later-phase female counterparts or males, we concluded that the most probable explanation was increased TRPM4 utilization during this phase. Using our genetically identifiable PLC*β*2-GFP+ Type II taste cells, we first confirmed the presence of TRPM4 in both males and females (Figure 3A). In patch-clamp experiments, we found that, like other GPCR-mediated tastants, TRPM4 was also an important factor in LA-induced activation of taste cells (Figure 3B,C). In both sexes, regardless of the estrous cycle phase, blocking TRPM4 resulted in a significant reduction in LA-evoked inward currents and depolarizations in Type II cells. This confirmed that, as with other tastants, TRPM4 plays an essential role in FA signaling in Type II cells. These data also suggest that early-phase females may be using a TRPM4- and TRPM5-*dependent* pathway. Together, these data show that TRPM4 is an essential part of the FA signaling pathway independent of sex and, most specifically, for the generation of LA-induced inward currents.

### 4.5. Early-Phase Females Demonstrate Enhanced Utilization of the TRPM5 Independent Pathway

To further understand the roles of both TRPM4 and TRPM5 in this FA signaling pathway, we employed ratiometric Ca^2+^ imaging. By perfusing the specific TRPM4 and TRPM5 antagonists, 9-PHE and TPPO, respectively, we directly observed the influence of each channel on LA-induced calcium responses (Figure 4). Inhibiting both TRPM4 and TRPM5 resulted in a nearly complete reduction in the LA-induced intracellular rise in calcium across both sexes and all stages of the estrous cycle. Although not significant, we observed a decrease in the magnitude of the response when the TRPM5 antagonist TPPO is used in both males and late-phase females, compared with the TRPM4 antagonists 9-PHE (Figure 4A,B). Early-phase females exhibit similar disruptions in calcium signaling when either TRPM4 or TRPM5 antagonists are applied (Figure 4C). This again suggests an increased reliance on TRPM4-activated pathways in Type II cells in the presence of higher endogenous estrogen.

To further test the role of TRPM4 in this fat-signaling pathway, we combined our *Trpm5* knockout mice with the TRPM4 antagonist 9-PHE. Interestingly, although trends were evident, males and late-phase females showed no significant difference in LA-induced calcium response magnitudes in the presence or absence of 9-PHE (TRPM4 antagonist) in *Trpm5*^−/−^ mice (see Figure 5). However, in Trpm5-deficient mice, early-phase females’ LA-induced calcium responses were greater and highly sensitive to 9-PHE. Thus, TRPM4 appears to play a greater relative role in fatty acid signaling under conditions of high circulating estrogen.

Together, these experiments have demonstrated that the FA signaling, and hence fat taste, is both sex and estrous cycle-dependent, and this dependence is tied to endogenous estrogen (E2) and its role in regulating FA activation in Type II cells. More specifically, in early-phase females, during high levels of endogenous E2, a TRPM5-*independent* pathway via TRPM4 plays a larger role than in males or in females with lower endogenous E2. Consistent with this, we observed a significant decrease in both inward current and cellular depolarization from females in the proestrus phase, suggesting that TRPM4 is the main channel used to activate LA responses in this high estrogen female group. We recently reported in taste cells that *Trpm4* expression is also estrous cycle-dependent, and the highest expression is observed when endogenous E2 is high [14]. Similarly, Eckstein and colleagues [44] found that TRPM4 displayed estrous-dependent function in the vomeronasal organ, another chemosensory organ, and may affect responses to olfactory cues; *Trpm4* is upregulated in cycling females and downregulated when the estrous cycle is abolished by ovariectomy. Estrogen receptors (ERs) are also present in taste cells [14]. Collectively, it is to be expected that TRPM4 significantly contributes to taste-induced activation in Type II cells, particularly in female mice.

## 5. Conclusions

Sex, a fundamental biological variable, has profound implications for physiology, behavior, and disease susceptibility [1,3,6,10,11,20,21]. Female mammals undergo cyclical hormonal fluctuations that significantly influence their physiological processes, including taste perception. Despite their implication in fat transduction of numerous tastants, the sex-specific involvement of TRPM channels in fat taste was not explored [16,23,25,26,27,28,29,31,32]. In this study, we explored the roles of TRPM4 and TRPM5 ion channels in mediating sex-specific responses to fat-taste cues. Our findings reveal that both channels play a critical role in fat taste detection in both sexes, but their activity is regulated by the estrous cycle. Given the implicit importance of TRPM channels in health and disease, these findings may shed light on estrogen’s influence on these channels in other physiological domains [35,36]. These results suggest that males and females may employ distinct signaling mechanisms to perceive and respond to dietary fat. This finding underscores a critical need for more highly focused sex-specific analyses in obesity and metabolic research. Specifically, the evidence for distinct signaling pathways suggests that all therapeutic interventions designed to combat metabolic syndrome and obesity must eventually account for these fundamental, sex-specific differences. Lack of consideration of sex within this research domain risks developing therapies that are highly effective in one sex but entirely inert or detrimental in the other.

## Data Availability

All relevant data are contained within the manuscript.

## Supplementary Materials

The following supporting information can be downloaded at https://www.mdpi.com/article/10.3390/nu17243847/s1, Figure S1: TRPM4 and IP_3_R3 are present in *Trpm5*^−/−^ mice.

## Abbreviations

The following abbreviations are used in this manuscript:

[Ca^2+^]_i_	Intracellular calcium
9-PHE	9-Phenanthrol/9-hydroxyphenanthrene
ATP	Adenosine triphosphate
BAPTA	1,2-bis(o-aminophenoxy)ethane-N,N,N′, tetraacetic acid
CT	Cycle threshold
DAPI	4′,6-diamidino-2-phenylindole
DIC	Differential interference contrast
E2	Estrogen
EGTA	Ethylene glycol-bis(β-aminoethyl ether)-N,N,N′,N′-tetraacetic acid
ER	Estrogen receptor
FA	Fatty acid
FURA2-AM	Fura-2 acetoxymethyl ester
GAD67	Glutamic acid decarboxylase 67
GPR120	G-protein coupled receptor-120
GTP	Guanosine triphosphate
HEPES	4-(2-hydroxyethyl)-1-piperazineethanesulfonic acid
IP_3_	Inositol 1,4,5-trisphosphate
IP_3_R3	Inositol 1,4,5-trisphosphate receptor type 3
LA	Linoleic acid
ns	Not significant
OCT	Optimal cutting temperature compound
P4	Progesterone
PFA	Paraformaldehyde
PIP_2_	Phosphatidylinositol 4,5-bisphosphate
PLC*β*2	Phospholipase C beta 2
PUFA	Polyunsaturated fatty acid
qRT-PCR	quantitative real-time reverse transcription polymerase chain reaction
ROI	Region of interest
TPPO	Triphenylphosphine oxide
TRC	Taste Receptor Cell
TRPM2	Transient Receptor Potential Melastatin 2
TRPM3	Transient Receptor Potential Melastatin 3
TRPM4	Transient Receptor Potential Melastatin 4
TRPM5	Transient Receptor Potential Melastatin 5
Trpm5^−/−^	Transient Receptor Potential Melastatin 5 Knockout
TRPM8	Transient Receptor Potential Melastatin 8
TRPMV4	Transient Receptor Potential Vanilloid 4
TRPV1	Transient Receptor Potential Vanilloid 1
TRPV6	Transient Receptor Potential Vanilloid 6
V_h_	Holding potential
Vmem	Membrane potential
