# Supplementary material for: Estrogen-Dependent Variation in the Contributions of TRPM4 and TRPM5 to Fat Taste
Source: Nutrients. 2025 Dec 10;17(24):3847. doi: 10.3390/nu17243847 (PMC12735415; doi:10.3390/nu17243847)
Supplement: Supplementary file 1 [file nutrients-17-03847-s001.zip › nutrients-4014839-supplementary.pdf]

nutrients

MDPI

Supplementary Material

Estrogen Dependent Variation in the Contributions of TRPM4 and TRPM5 to Fat Taste

Emeline Masterson[1,2], Naima S. Dahir[1,2], Ashley N. Calder[1,2], Yan Liu[2], Fangjun Lin[1,2], and Timothy A. Gilbertson [2,*]

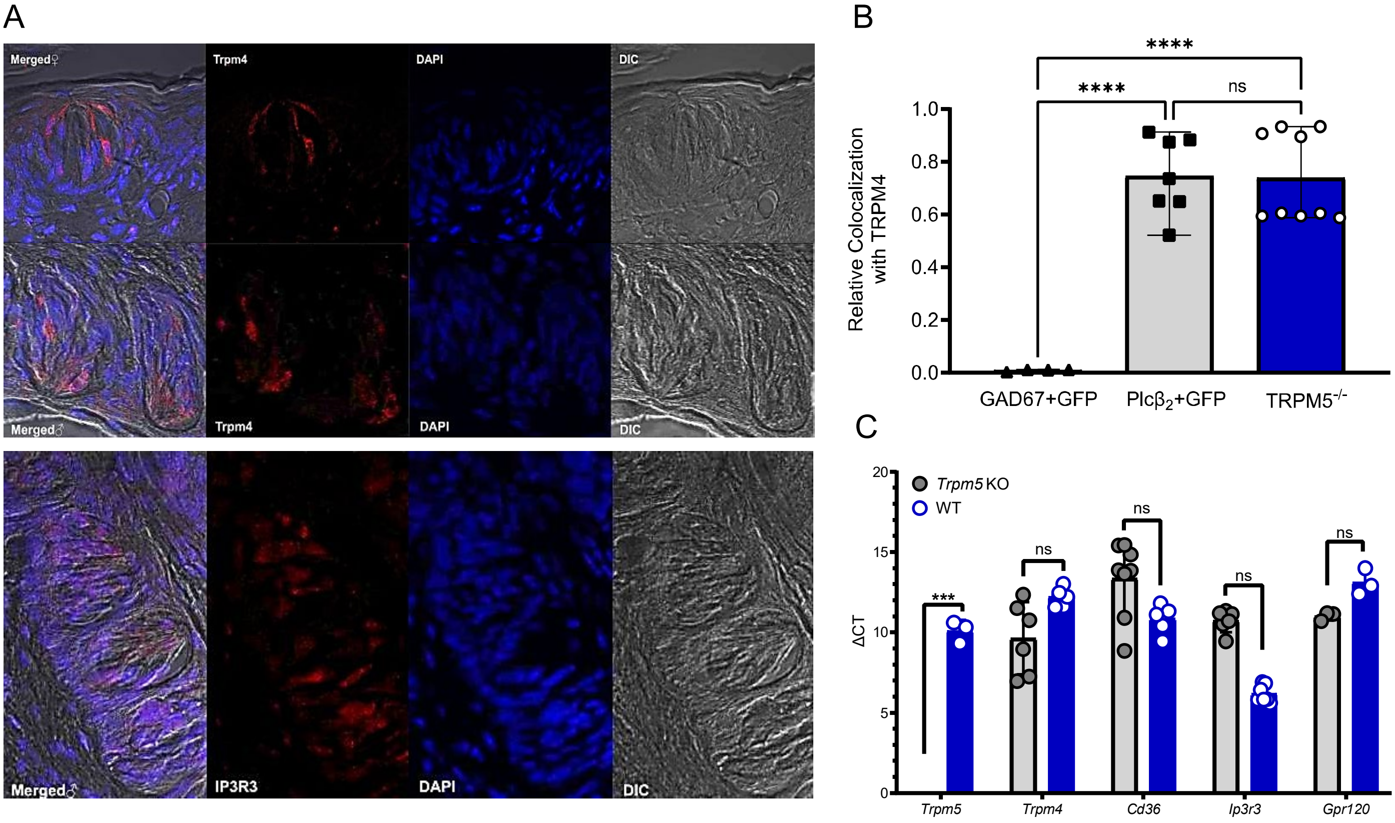

Figure S1. TRPM4 and IP_3R3 are present in *Trpm5*$^{-/-}$ mice. (A) Immunohistology images from male and female knockout mice indicate the presence of TRPM4 (upper panels) and IP_3R3, shown for male mice (lower panels). Image groups denote merged, primary antibody (TRPM4 or IP_3R3), DAPI, and differential contrast images (DIC) using Nikon confocal microscopy. **(B)** Manders' colocalization coefficients for TBCs in GAD67 (Type III cells), PLCβ_2 (Type II cells), and *Trpm5*$^{-/-}$ mice were determined using NIS-elements image analysis. Relative correlation coefficients are calculated by defining Regions of Interest (ROIs), which are delineated around target structures, such as taste buds, along with establishing standard intensity thresholds to minimize background noise. Subsequently, the software calculates the relevant colocalization coefficients within the designated ROIs. Mander's coefficients are calculated by summing the intensities of pixels in one channel only, where the intensity of the other channel is above zero, and then dividing by the total intensity of the respective channel. Mander's coefficients allow for an assessment of how much of each protein or structure is found in the presence of the other target. These coefficients provide a robust, quantitative assessment of the degree to which each target protein or structure is found in the presence of the other. **(C)** Delta C_T (cycle threshold) values measured from qRT-PCR utilizing knockout and wild-type mice, asterisks denote no significant differences between WT and KO individuals from 2-way ANOVA with Tukey's post-hoc multiple comparisons. As expected, *Trpm5* was not detectable in taste cells from *Trpm5*$^{-/-}$ mice. Asterisks denote significant differences in ordinary one-way ANOVA with multiple comparisons: *** $p < 0.001$, **** $p < 0.0001$.
